# Kynurenine monooxygenase *BcKMOL*: a key regulator of growth, pathogenicity, and disease control in *Botrytis cinerea*

**DOI:** 10.3389/fmicb.2025.1595008

**Published:** 2025-06-24

**Authors:** Bai Li, Xiaoying Liu, Jinping Zang, Hongzhe Cao, Helong Si, Kang Zhang, Jihong Xing, Jingao Dong

**Affiliations:** ^1^State Key Laboratory of North China Crop Improvement and Regulation, Hebei Agricultural University, Baoding, China; ^2^Hebei Key Laboratory of Plant Physiology and Molecular Pathology, Hebei Agricultural University, Baoding, China

**Keywords:** *Botrytis cinerea*, kynurenine pathway, kynurenine monooxygenase, growth and pathogenesis, antimicrobial peptides

## Abstract

Kynurenine monooxygenase, a vital rate-limiting enzyme in the kynurenine pathway metabolic branch, has shown promise as a drug target for treating human neurodegenerative diseases. However, the role of kynurenine monooxygenase in plant pathogens and its potential as a molecular target have received limited attention. In this study, we identified a novel kynurenine monooxygenase gene, *BcKMOL*, in *Botrytis cinerea*. By generating mutants of this gene, it was found that the deletion of *BcKMOL* affected the changes of key metabolites in the kynurenine pathway *in vivo*, and the △*BcKMOL* mutant exhibits reduced growth and fails to produce sclerotia. Additionally, changes were observed in the morphology of mycelium cells and spores, and the mutant’s pathogenicity was weakened. These findings indicate that *BcKMOL* positively regulates the growth, development, and pathogenic processes of *B. cinerea*. Furthermore, we screened two antibacterial peptides, CAMPQ3966 and CAMPQ4589, that target *BcKMOL* using MEGADOCK, HDOCK, and AlphaFold3. Both peptides effectively inhibited the pathogenicity of *B. cinerea*. These findings provide the foundation for developing novel drug targets for controlling gray mold.

## 1 Introduction

*Botrytis cinerea*, commonly known as the “gray mold fungus,” is responsible for causing significant pre- and post-harvest diseases in a wide range of plant species (Carrasco et al., 2017). As a typical necrotrophic plant pathogenic fungus, it can affect nearly a thousand different crops (Zhu et al., 2022). *B. cinerea* can form a black dormant structure called sclerotium during winter or under unfavorable conditions. Secondary inoculant sources include sclerotia, diseased straw, and infected seeds (Richardson et al., 2011). The fungus primarily spreads through conidia in humid environments with cool and optimal temperatures ranging from 15 to 23°C (Hua et al., 2018; Lecompte et al., 2017). The ubiquity of its conidia in the air leads to substantial losses (Plesken et al., 2021).

In recent years, various strategies have been proposed to manage *B. cinerea* infections and improve the postharvest management of fresh melons and fruits, thereby preventing quality degradation (Ceredi et al., 2009; Lichter et al., 2016; Sonker et al., 2016). Currently, the prevention and control of gray mold primarily rely on fungicides. However, the extensive use of fungicides has resulted in multiple drug-resistant strains of *B. cinerea*, which exhibit resistance to various fungicide groups (Yan et al., 2022). The overuse of fungicides significantly negatively impacts human health (Muri et al., 2009), soil microflora (Meena et al., 2020), and beneficial microorganisms in food and beverage fermentation (Pasquale et al., 2019). Therefore, it is crucial to delve deeper into the pathogenic mechanisms of *B. cinerea* and explore practical and safe long-term treatment options for this fungus.

The emergence of antimicrobial peptides (AMPs) opens up a green, safe and new path for gray mold control. AMPs are a class of small molecular peptides ubiquitous in nature and an integral part of the innate immunity of almost all organisms (Li et al., 2022). Certain AMPs exert antifungal effects in fungi by inhibiting the synthesis of fungal cell wall components, such as glucan, chitin, and glycoprotein (Li et al., 2021). For instance, echinocandin, a glucan inhibitor, acts as a noncompetitive inhibitor of β-(1,3)-glucan synthase, affecting fungal cell wall synthesis. The cell wall is a promising target for AMPs to recognize microbial cells. AMPs targeting the cell wall primarily exert antibacterial effects by disrupting the synthesis and structure of cell wall components (Wasmann et al., 2018).

The kynurenine pathway is the primary route of tryptophan metabolism, profoundly influencing numerous biological processes within organisms (Davidson et al., 2022). A key terminal product of this pathway, NAD(P)^+^, serves as a crucial cofactor in redox reactions occurring *in vivo*. Notably, in certain microorganisms, the kynurenine pathway is the sole means of NAD^+^ biosynthesis (Lima et al., 2009), and it is closely related to many life processes such as energy metabolism, gene expression regulation, and secretion (Xie et al., 2020). In fungi and bacteria, siderophores are essential for synthesizing cytochromes and enzymes. Tryptophan-2,3-dioxygenase (TDO), a key rate-limiting enzyme within the kynurenine pathway, is closely associated with synthesizing siderophores. Studies have shown that deletion of TDO in *Pseudomonas* affects siderophores synthesis, and exogenous addition of kynurenine can restore it (Matthijs et al., 2004). Further along the pathway, L-kynurenine is converted to 3-hydroxy-L-kynurenine by either flavinase or kynurenine monooxygenase. This compound serves as an essential precursor for the production of quinone antibiotics (Hirose et al., 2011). For instance, the actinomycin biosynthetic gene cluster in *Streptomyces chrysomallus* includes the corresponding enzyme genes of the kynurenine pathway. The enzymes encoded by these genes facilitate the generation of an intermediate product via the kynurenine pathway, which subsequently provides the precursor 4-methyl-3-hydroxyanthranilic acid (4-MHA) for actinomycin biosynthesis (Keller et al., 2010).

Kynurenine monooxygenase (KMO) serves as a rate-limiting enzyme at the pivotal branch point within the kynurenine pathway, located on the outer membrane of mitochondria (Amaral et al., 2013; Giorgini et al., 2013; Wilson et al., 2014). It regulates the breakdown of the neuroactive metabolite kynurenine (KYNU) and 3-hydroxykynurenine (3-HK) synthesis. In recent years, KMOs have received considerable attention as drug targets in neurodegenerative diseases research. The crystal structure of *Saccharomyces cerevisiae* ScKMO was first elucidated in 2013 (Hughes et al., 2022). Subsequently, it was reported that PfKMO from *Pseudomonas fluorescens* is a soluble enzyme with a structure similar to that of ScKMO (Phillips et al., 2019). The C-terminal domain of PfKMO is crucial for substrate binding. In the absence of a substrate or inhibitor, PfKMO adopts an ‘open’ conformation, facilitating the rapid binding of substrates or inhibitors and the release of products. Upon binding, the C-terminal domain conforms to a ‘closed’ state, effectively inhibiting KMO activity (Gao et al., 2018; Wilkinson, 2013). The human-specific inhibitor UPF648 (Hughes et al., 2022) exploits this mechanism to inhibit KMO, redirecting kynurenine pathway metabolism to enhance the production of the neuroprotective compound kynurenic acid and ameliorate disease-related phenotypes (Amaral et al., 2013).

In this study, we identified the kynurenine monooxygenase gene *BcMKOL* in *B. cinerea* through bioinformatics analysis. We then elucidated the role of *BcKMOL* in growth, development, and pathogenicity by constructing knockout mutants of △*BcMKOL*. Furthermore, we utilized MEGADOCK, HDOCK, and AlphaFold3 to screen and identify antibacterial peptides that effectively inhibit the pathogenicity of *B. cinerea*. Our research findings provide valuable insights and innovative ideas for the green, safe, and sustainable prevention and control of gray mold.

## 2 Materials and methods

### 2.1 Strains and cultural conditions

The wild-type strain B05.10 of *B. cinerea* was preserved in the Key Laboratory of Plant Physiology and Molecular Pathology of Hebei Province. Mycelia were inoculated in a PD medium (PDA medium without agar) and cultured at 25°C for 5 days for RNA and DNA extraction. For protoplast preparation, the wild-type strain was inoculated on PDA medium for 7–10 days, and the mycelium was scraped using Fries medium and cultured at 25°C to obtain a conidial suspension.

### 2.2 Bioinformatics analysis of kynurenine monooxygenase gene *BcKMOL*

The whole genome sequence of B05.10 was searched in the Ensembl fungi database using the amino acid sequence of human and mouse kynureninease. Multiple sequence alignment was performed to analyze the results, and the protein’s three-dimensional structure was predicted using the Swiss Model.^1^ Expression profile data of the *B. cinerea* infection process was obtained from the GEO^2^ database, and the expression data of the *KMO* gene at different times points were compared to the control (PRJNA628162). The log2 value were taken, and the expression level differences in each pathogen infection period were analyzed.

### 2.3 Construction of *BcKMOL* knockout mutants

Based on the restriction enzyme digestion site of the *BcKMOL* gene and the multiple cloning site of the pBS-pUC vector, Snapgene was used to find a suitable restriction enzyme digestion site. Gene-specific primers containing the restriction enzyme digestion site were designed (Table 1), and the target fragment was amplified. After digestion and ligation into the pMD19 cloning vector, positive clones with correct sequencing were ligated into pUC, and the positive clones were verified. The knockout mutant vector was used to obtain the knockout mutant △*BcKMOL* by protoplast transformation. DNA level analysis, Southern Blot, and qRT-PCR were performed to confirm the knockout.

**Table 1:** The primers sequence.

Gene name	Primer name	Sequence (5′–3′)
*BcKMOL*I	*BcKMOL*I-F	GAGCTCTGTTTGGTACGGCTTTAGAAT
	*BcKMOL*I-R	ACTAGTGCGAGGAACTCCATCTCTCCA
*BcKMOL*II	*BcKMOL*II-F	CTCGAGCATAAGGGTCGTTAAATGAGC
	*BcKMOL*II-R	GGTACCTGTCAAGTCGAAGGAGCACA
pEarleyGate104-*BcKMOL*	pEarleyGate104-*BcKMOL*-F	CACCATGTCTGGACAATCTTCAAGACA
	pEarleyGate104-*BcKMOL*-R	AGCAGTCTTCCATCCCAATAATGCT
*BcKMOL* hygromycin B	P1	CAACATGTTTGAAGCTTGGCAC
	P2	GGAGCAGCAGACGCGCTA
*BcKMOL* hygromycin B	P3	CTGCAGAACAGCGGGCAG
	P4	GACATGTTCATCCTAGACAATTGG
hygromycin B	P5	CTATTCCTTTGCCCTCGGA
	P6	ATGAAAAAGCCTGAACTCACCGC
*BcKMOL*	P7	TCATCCCCTCGTTGAGCTG
	P8	ATTGTAAAGAAGAGCCAGAAACAGG
Rt*BcKMOL*	Rt*BcKMOL*-F	CGACACTCTGTGGTGTGAGT
	Rt*BcKMOL*-R	TCGAGCTCATTGAACGGCTT
KanR	KanR-F	CTCCCAATCAGGCTTGATCCC
	KanR-R	ATGGCTAAAATGAGAATATCACCGG
GLF	GLF-F	TCAAATCTGGTGACGGGCAGGAC
	GLF-R	ATGAGCCCAGAACGACGC
*Bcpg1*	*Bcpg1*-rt-F	ATGGTTCAACTTCTCTCAATGGC
	*Bcpg1*-rt-R	TGGGACGGAGAGTGCGCTGAGGACG
*Bcpg5*	*Bcpg5*-rt-F	ATGGTTAAGTTTTCTGCCTGTC
	*Bcpg5*-rt-R	GCCAGATGGGACTGCAAG
*Bcpgx1*	*Bcpgx1*-rt-F	ATGCATTTTCAATTGAGC
	*Bcpgx1*-rt-R	GGTTCTAGCTGCCGGAGAAGGTGCA
*Bcpg4*	*Bcpg4*-rt-F	ATGCCTTCCACCAAGTCCA
	*Bcpg4*-rt-R	GATCGGTCATGTCAAGGGTGACACC
*Bcpg3*	*Bcpg3*-rt-F	ATGCGTTCTGCGATCATCCTC
	Bcpg3-rt-R	TTGAAGACAACTGCATGCACTAGAC
*Bcpme2*	*Bcpme2*-rt-F	ATGCGTTCCTTTGCCCTCCTCTCCC
	*Bcpme2*-rt-R	ATCGGTGGTGGTGGTGGACAATGCA
*Bcmnl1*	*Bcmnl1*-rt-F	ATGGAGCTGGCATTCCCGGA
	*Bcmnl1*-rt-R	TTGAAGTGCTGTTGCGCCGGTTTCT
*Bcams1*	*Bcams1*-rt-F	ATGGGTGGTGAAACTGTCTTA
	*Bcams1*-rt-R	CGATAGTTTGATGTGCGGTTCTCCA
*Bcpme1*	*Bcpme1*-rt-F	ATGCCGCAGTTCAGAGGAAGCCC
	*Bcpme1*-rt-R	TTGGTATCCGATGAACTGGGTGGCA
*BccutA*	*BccutA*-rt-F	ATGAAGACCTCAGCTCAACAAC
	*BccutA*-rt-R	GGTGACTGAAGATGACCCAAGAGCA
*BccutB*	*BccutB*-rt-F	ATGAAGTTTTCACAGTCTCTG
	*BccutB*-rt-R	GGCAAAGATAAGCATCATTGGTGCG
*Bcmns1*	*Bcmns1*-rt-F	ATGAACAGTGCGACCCCTTTAACC
	*Bcmns1*-rt-R	CAAATTGCGCTGAGGATCTGTGAAA
*β-Tublin*	*β-Tublin*-F	GAGCGTGAAATCGTCCGTG
	*β-Tublin*-R	GGATACCACCGCTCTCAAGAC

The genomic DNA of *B. cinerea* B05.10 wild-type and knockout transformants △*BcKMOL* was extracted, and single enzyme digestion was performed using the restriction enzyme *Xba* I. The enzyme-digested genomic products were detected using 0.8% agarose gel to ensure that the enzyme digestion was sufficient. After purification, the purified product was separated by overnight electrophoresis with 0.8% agarose gel, transferred to the membrane, and the hygromycin resistance gene was labeled with digoxin (Zhang et al., 2025) to make a probe for overnight hybridization (Supplementary Table S1). The specific steps were performed according to the (Roche) kit.

### 2.4 Construction of *BcKMOL* revertant mutant

Snapgene was used to analyze the pEarleyGate104 expression vector and the CDS sequence of the *BcKMOL* gene. Specific primers (*BcKMOL*-pEarleyGate104-F/*BcKMOL*-pEarleyGate104-R; Table 1) were designed to amplify the full-length gene, and the correct positive clone was named pEarleyGate104-*BcKMOL*. *BcKMOL*-C transformants were obtained by ATMT transformation. PCR using the glufosinate gene on the vector and gene-specific primers was used to identify the transformants. Real-time PCR was performed to confirm the transformants, using the *Tubulin* gene as the internal reference.

### 2.5 Kynurenine pathway metabolites analysis of the gene *BcKMOL* gene mutants

The wild-type and mutant △*BcKMOL* were inoculated in a liquid PD medium, respectively, and grew for 10 days at 25°C in the dark. After treatment with a freeze-dryer, 80% methanol was mixed and placed in a 2 mL adapter. The liquid nitrogen was frozen for 5 min, repeated freezing and thawing twice, and 900 μL 10% methanol was added. The liquid nitrogen was repeatedly frozen and thawed and installed in a freeze grinder. Centrifuge at 4°C for 5 min at 12,000 rpm/min. 100 μL supernatant was added to 100 μL 20 ppb Trp-d5 solution and vortexed for 10 s. 150 μL supernatant was added to the detection bottle, and the contents of tryptophan, kynurenine, 3-hydroxy kynurenine and quinolinic acid, the key metabolites of kynurenine pathway, were determined by tryptophan targeted metabolic analysis.

### 2.6 Growth and development analysis of the *BcKMOL* gene mutants

The *BcKMOL* gene mutants were inoculated on the non-resistant PDA medium to observe the colony growth rate, recorded every 24 h. Conidia growth of the *BcKMOL* gene mutants were observed. Five-day-old mutant colonies were rinsed with 5 mL sterile ddH_2_O, and 10 μL was placed on a slide for microscopic observation. Mycelium morphology was observed under a fluorescence microscope after CFW staining, and cell length and width were counted.

### 2.7 Pathogenicity analysis of the *BcKMOL* gene mutants

Tobacco leaves were surface-disinfected with 75% alcohol for 3 min and washed with sterile water 2–3 times. Uniform fungal plates were made using an 8 mm puncher from 7-day-old mutant and wild-type strains. Colonies were inoculated on tobacco leaves using 50% Tween drops and moisturized under dark conditions at 25°C. Disease incidence was observed, and lesion areas were statistically analyzed using a multi-spectral scanner. Three replicates were set for each strain, and one-way ANOVA was performed using GraphPad software (significant: *: 0.01 < *p* ≤ 0.05; highly significant: **: *p* ≤ 0.01).

### 2.8 Analysis of pathogenic factors of the *BcKMOL* gene mutants

The *BcKMOL* gene mutants were inoculated on the non-resistant PDA medium, and infection pads were observed under the microscope. The number and size of the infection pads were statistically analyzed. The *BcKMOL* gene mutants and wild-type strains were inoculated on a PDA medium containing 0.05% bromothymol blue and incubated in complete darkness at 22°C for 7 days to assess acid secretion. Cell wall-degrading enzymes activity was determined using an enzyme activity detection kit after filtering the culture medium of 14-day-old liquid PD cultures. qRT-PCR analyzed the expression levels of cell wall-degrading enzyme-related genes in the *BcKMOL* gene mutants.

### 2.9 Screening of antimicrobial peptides targeting *BcKMOL*

The three-dimensional structure of *BcKMOL* was constructed, and docking software was used to analyze the target protein sequence. MEGADOCK was used to predict eigenvalues, and the protein–protein interaction data with antibacterial peptides were obtained. The top 200 protein groups were selected based on the MEGADOCK score and docked in batches using HDOCK docking software. The top 10 combinations were selected for AlphaFold3 interaction analysis.

### 2.10 Simulated molecular docking of *BcKMOL* and antimicrobial peptides

The protein three-dimensional structure of *BcKMOL*, CAMPQ3966, and CAMPQ4589 was established using Swiss-Model,^3^ and molecular simulation docking was performed using HDOCK SERVER.^4^ The results were imported into Pymol for in-depth analysis (Wang et al., 2024).

### 2.11 Induced expression and purification of antimicrobial peptides

The two antimicrobial peptides with the highest docking scores were selected as CAMPQ3966 and CAMPQ4589. After codon optimization, the prokaryotic expression vectors were constructed respectively, and the plasmids were extracted and transformed into *E. coli*BL21. The positive clones pET28a-*CAMPQ3966* and pET28a-*CAMPQ4589* were inoculated into 20 mL LB medium containing kanamycin sulfate and cultured overnight. The overnight culture broth was taken at a ratio of 1:20 and inoculated into LB medium preheated to 37°C and containing kanamycin sulfate. Conventional culture at 37°C for about 30–60 min or longer, until the OD600 of the bacterial solution reached 0.5–0.7; the pET28a-*CAMPQ3966* strain was added with IPTG to a final concentration of 0.1 mM and induced at 16°C for 24 h. The pET28a-*CAMPQ4589* strain was added with IPTG to a final concentration of 1 mM and induced at 28°C for 12 h. The bacterial liquid was collected into a centrifuge tube, centrifuged at 4°C, 15,000 *g* for 1 min, discarded the supernatant, and collected the precipitate; the supernatant was resuspended with Tris–HCL, added 5 × louding buffer, boiled for 10 min, centrifuged at 12,000 *g* for 3 min, and 20 μL of the supernatant was placed on 10% SDS-PAGE gel electrophoresis, 80 V electrophoresis for 1 h, 100 V electrophoresis for 1 h, Coomassie brilliant blue staining for 15 min, washed with water, and observed protein induction.

The antimicrobial peptide CAMPQ3966 was induced to express at 16°C for 24 h, and CAMPQ4589 was induced to express at 28°C for 12 h. Antibacterial peptides CAMPQ3966 and CAMPQ4589 were induced in large quantities and purified using an AKTA protein purification instrument after ultrasonic lysis and centrifugation.

### 2.12 Effect of antimicrobial peptide on the growth and development of *B. cinerea*

The wild-type strain of *B. cinerea* and the *BcKMOL* mutants at the same growth period were inoculated on PDA solid plates. After 5 days of dark culture at 25°C, a bacterial plate with a diameter of 5 mm was punched with a sterile puncher for subsequent inoculation experiments. The same concentration of CAMPSQ3966 and CAMPSQ4589 proteins were coated on the right side of the culture dish, and the same volume of protein eluent was coated on the left side of the culture dish as the control group. The wild-type strain of *B. cinerea* and the *BcKMOL* mutants were inoculated to observe the growth of the strain.

### 2.13 Effect of antimicrobial peptide on pathogenicity of *B. cinerea*

The wild-type strain B05.10 of *B. cinerea* was inoculated on a PDA solid plate and cultured for 5 days. A 5 mm diameter plate was punched for subsequent inoculation experiments. *N. benthamiana* plants at 4–5 weeks were selected. CAMPQ3966 and CAMPQ4589 proteins were applied to the right side of tobacco leaves, with soluble Elution Buffer applied to the left side as a control. Elution Buffer was prepared from 5.844 g NaCl, 4 mL 20 mM Tris–HCl, 100 mL 1 M Imidazole, and diluted to 200 mL. After 24 h, leaves were inoculated with B05.10, and plants were placed in a humid environment at 25°C for dark culture. Tobacco leaf disease incidence was observed and photographed after 2 days of inoculation. The same number of diseased leaves were taken and RNA was extracted. The *Tublin* gene was used as the reference gene of *Botrytis cinerea*, and the fungal biomass during the disease period was detected by qRT-PCR. The protein CAMPSQ3966 and CAMPSQ4589 were mixed with Tween in equal proportions, and an appropriate amount was dropped on the surface of apples or pears. In the control group, the protein eluent was mixed with Tween in equal proportions, and an appropriate amount was added to the other side of the same apple or pear. The wild-type strain of *B. cinerea* at the same growth period was inoculated and placed in dark to observe the incidence.

### 2.14 qRT-PCR

RNA was extracted from the knockout transformants and wild-type strains, and reverse transcribed into cDNA. The expression of the target gene in the transformants was detected by Real-time PCR using *Tubulin* gene as an internal reference (Supplementary Table S1). The reaction system was: template (cDNA) 2.0 μL, Mix (5 U/μL) 10.0 μL, forward primer (10 μM) 0.4 μL, reverse primer (10 μM) 0.4 μL, ROX 1.0 μL, ddH_2_O 6.2 μL, the total system was 20 μL. The reaction procedure was carried out in strict accordance with the Takara fluorescence quantitative RT-PCR kit.

### 2.15 ELISA

The appropriate amount of mycelium or leaf lesion site was taken, grinded and crushed, diluted with PBS buffer solution with pH = 7.2, after repeated freezing and thawing, the ultrasonic crushing instrument was used for crushing. Centrifuging at 4°C, 2,000 rpm/min for 20 min, and the supernatant was carefully collected. The kynurenine monooxygenase activity of BcKMO protein was determined by the Human KMO ELISA kit (DLDEVELOP, Canada). The specific steps were carried out according to the instructions of the kit. The linear regression equation of the standard curve was calculated by using the concentration and OD value of the standard substance, and the OD value of the sample was substituted into the equation to calculate the enzyme activity of the sample.

## 3 Results

### 3.1 *BcKMOL* encodes kynurenine monooxygenase in *B. cinerea*

**Figure 1:**
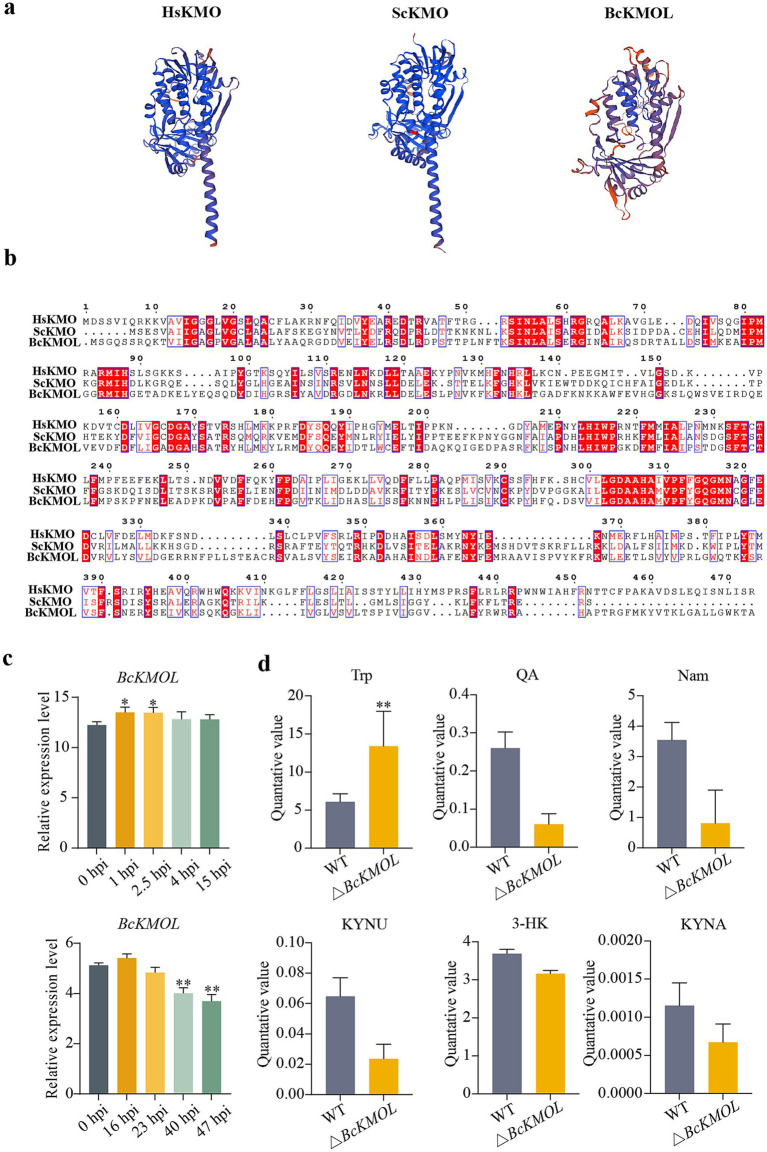
Bioinformatics analysis of kynurenine monooxygenase KMO. **(a)** Advanced structural analysis. A phylogenetic tree and domain of *B. cinerea* KMO and its human homologous were generated using the Swiss Model. **(b)** Multiple sequence alignment. Protein sequences of HsKMO (human), ScKMO (yeast), and *BcKMOL* (*B. cinerea*) were aligned using MEGA software. **(c)** The expression level of *BcKMOL* in each stage of conidial growth and infection of *B. cinerea* was analyzed. The expression level of *BcKMOL* during the growth and development of *B. cinerea* conidia was analyzed at 0 hpi. The expression level of *BcKMOL* during *B. cinerea* infection was analyzed at 0 hpi when tomato was infected. **(d)** Analysis of kynurenine pathway metabolites in *BcKMOL* mutants. △*BcKMOL-1*, △*BcKMOL-2,* and △*BcKMOL-3* are three biological replicates. Each histogram represents the average SD from three biological replicates, and the asterisk indicates a significant difference from WT, *p < 0.05, **p < 0.01.

Utilizing the amino acid sequence of kynurenine monooxygenase (KMO) from humans and yeast, a search was conducted in the Ensembl Fungi database for the corresponding sequence in *B. cinerea* B05.10 strain. The search revealed that *Bcin01g01500,* henceforth referred to as *BcKMO*-like or *BcKMOL*, exhibited high similarity to KMO in other species. The Swiss-Model website was used to analyze the advanced structure of *BcKMOL* and found that it was similar to the structure of other KMOs (Figure 1a). Multiple sequence alignment was performed using MEGA7.0 software, which highlighted several highly conserved sequences between *BcKMOL*, HsKMO (human KMO) and ScKMO (yeast KMO) (Figure 1b). By analyzing the expression profile data of the wild-type *B. cinerea* strain from the GEO database during its growth, development, and infection phases, it was observed that the expression level of the *BcKMOL* gene increased within the first 1–15 h post-inoculation (hpi) during pathogen growth and development. Notably, the expression level peaked at 16 hpi during pathogen infection but gradually declined thereafter (Figure 1c). These findings suggest that *BcKMOL* plays a role in the growth and development of *B. cinerea*. By detecting the activity of *BcKMOL* at 16 hpi, the enzyme activity of *BcKMOL* was significantly higher than that of uninfected (Supplementary Figure S3a).

To elucidate the impact of *BcKMOL* on growth and development of *B. cinerea*, we successfully generated knockout mutants and revertant strains of the *BcKMOL* gene (Supplementary Figures S1, S2). It was found that the deletion of *BcKMOL* affected the content of key metabolites of KP, in which the upstream product tryptophan was significantly accumulated, and the downstream metabolites 3-HK and QA were decreased. The content of other key metabolites such as KYNU, KYNA, and Nam also changed (Figure 1d). The contents of metabolites related to the other two pathways of tryptophan metabolism: the bacterial degradation pathway and the 5-hydroxytryptamine pathway also changed (Supplementary Figure S3b). It is speculated that *BcKMOL* encodes kynurenine monooxygenase and regulates the growth, development, and infection of *B. cinerea.*

### 3.2 *BcKMOL* affects the growth and development of *B. cinerea*

**Figure 2:**
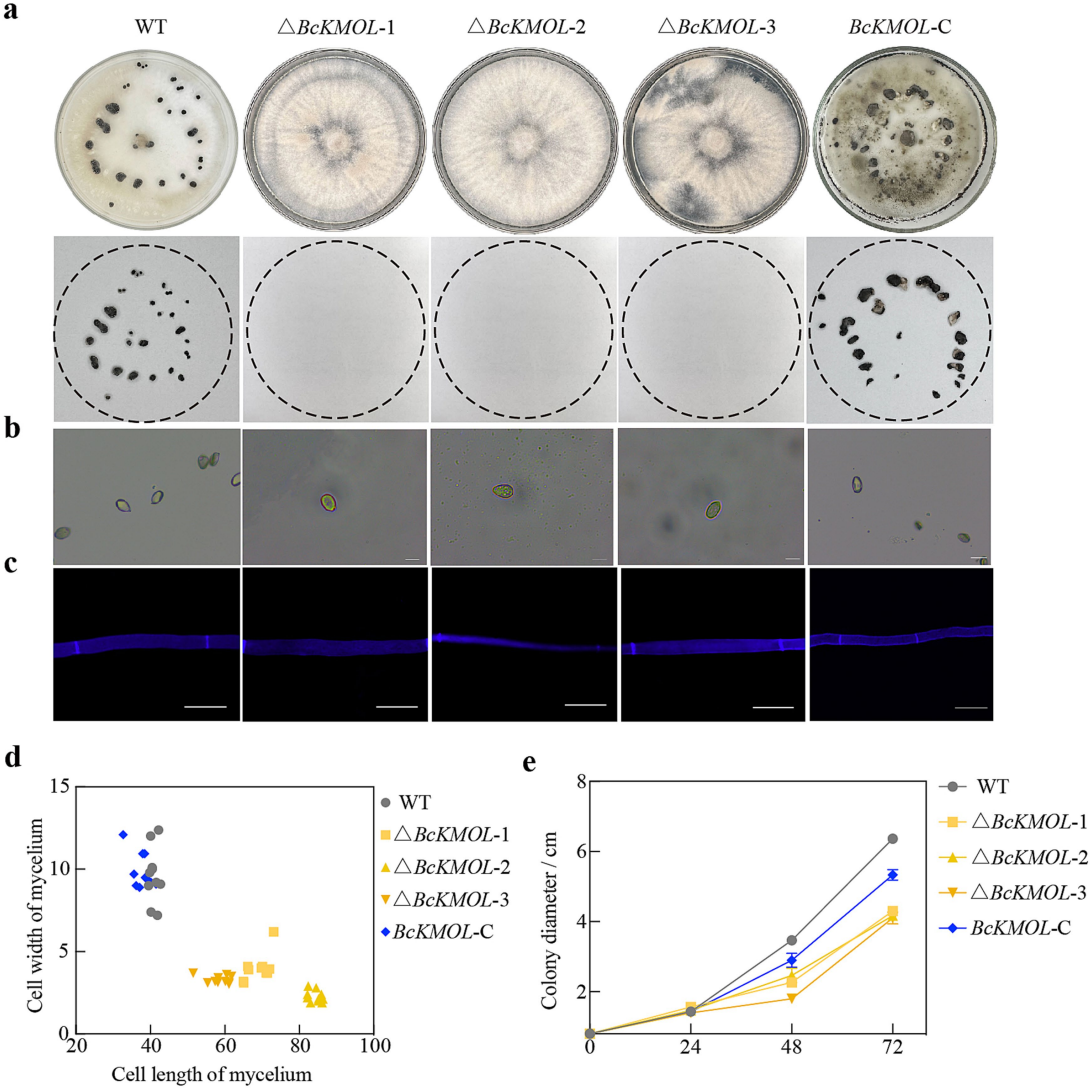
Role of the *BcKMOL* gene in *B. cinerea* growth and development. **(a)** Sclerotia growth of analysis. The growth of sclerotia in the *BcKMOL* gene mutants on the non-selective PDA medium was observed and recorded. **(b)** Spore morphological observation. Colonies were washed, filtered, and observed under a microscope. Scale bar: 10 μm. **(c)** Mycelial morphology observation. Mycelia stained with Calcofluor White (CFW) were observed under a fluorescence microscope. Scale bar: 20 μm. **(d)** Mycelial morphology analysis. A box plot representing the width and length of mycelial cells, used for growth rate analysis of the *BcKMOL* gene mutants. **(e)** Growth rate statistics of *BcKMOL* gene mutants. The *BcKMOL* gene mutants and wild type at the same growth stage were inoculated on PDA plates, and the colony diameter was recorded every 24 h.

We then observed the colony morphology, mycelial morphology, and growth rate of these *BcKMOL* gene mutants (Figure 2). Our findings revealed that the knockout mutants of the *BcKMOL* gene failed to produce sclerotia, and their conidia exhibited an elongated morphology compared to the wild type. Furthermore, the mycelial cells of the mutants were significantly longer and considerably narrower than those of the wild type. Notably, the *BcKMOL*-C mutant exhibited a substantial restoration in sclerotium production, spore morphology, mycelial cell length, and growth rate when compared to the wild type. These results collectively suggest that mutations in the *BcKMOL* gene exert a specific influence on the growth and development of *B. cinerea*, thereby implicating the *BcKMOL* gene in the regulation of the growth and development processes in *B. cinerea*.

### 3.3 *BcKMOL* positively regulates the pathogenicity in *B. cinerea*

**Figure 3:**
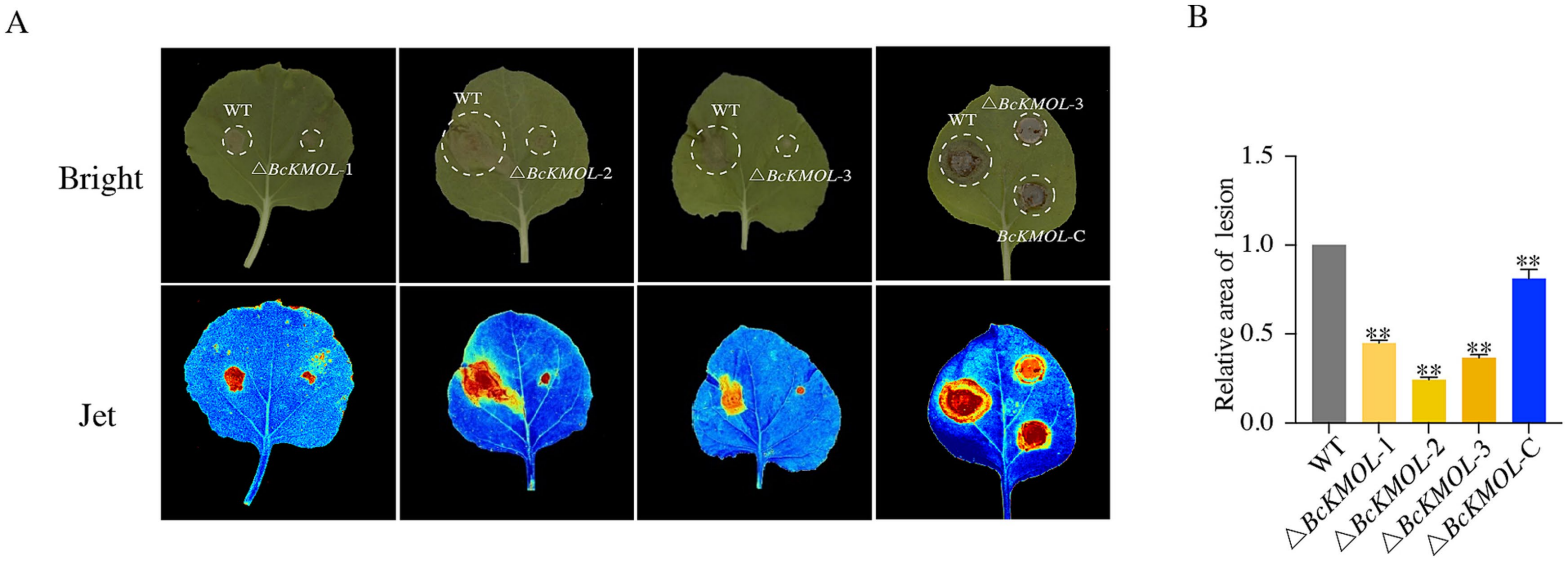
Pathogenicity analysis of the *BcKMOL* gene mutants. **(A)** Pathogenicity analysis. Tobacco leaves were inoculated with the *BcKMOL* gene mutants and wild-type strains of the same growth period. The mutants and wild type at the same growth stage were inoculated on tobacco leaves, placed in a humid environment at 25°C, and treated in the dark for 48 hpi to analyze the lesion area. Each group of treatments had three biological replicates. Bright denotes standard optical mode, and Jet denotes multi-spectral imaging mode. **(B)** Lesion area statistics. Error bars represent the standard deviation from three biological replicates. Asterisks indicate significant differences from the wild type (WT) *p < 0.05, **p < 0.01.

To investigate the role of the *BcKMOL* gene in the pathogenesis of *B. cinerea*, we inoculated tobacco leaves with both the wild type and the *BcKMOL* gene mutants at the same growth stage. The results showed that while the △*BcKMOL* mutant was capable of producing visible lesions on tobacco leaves, the lesion area was significantly smaller compared to that caused by the wild type (Figure 3). Moreover, the pathogenicity of the *BcKMOL*-C revertant strain was restored, confirming that the *BcKMOL* gene exerts a positive regulatory effect on the pathogenesis of *B. cinerea*.

### 3.4 *BcKMOL* impacts infection cushion formation and acid production in *B. cinerea*

**Figure 4:**
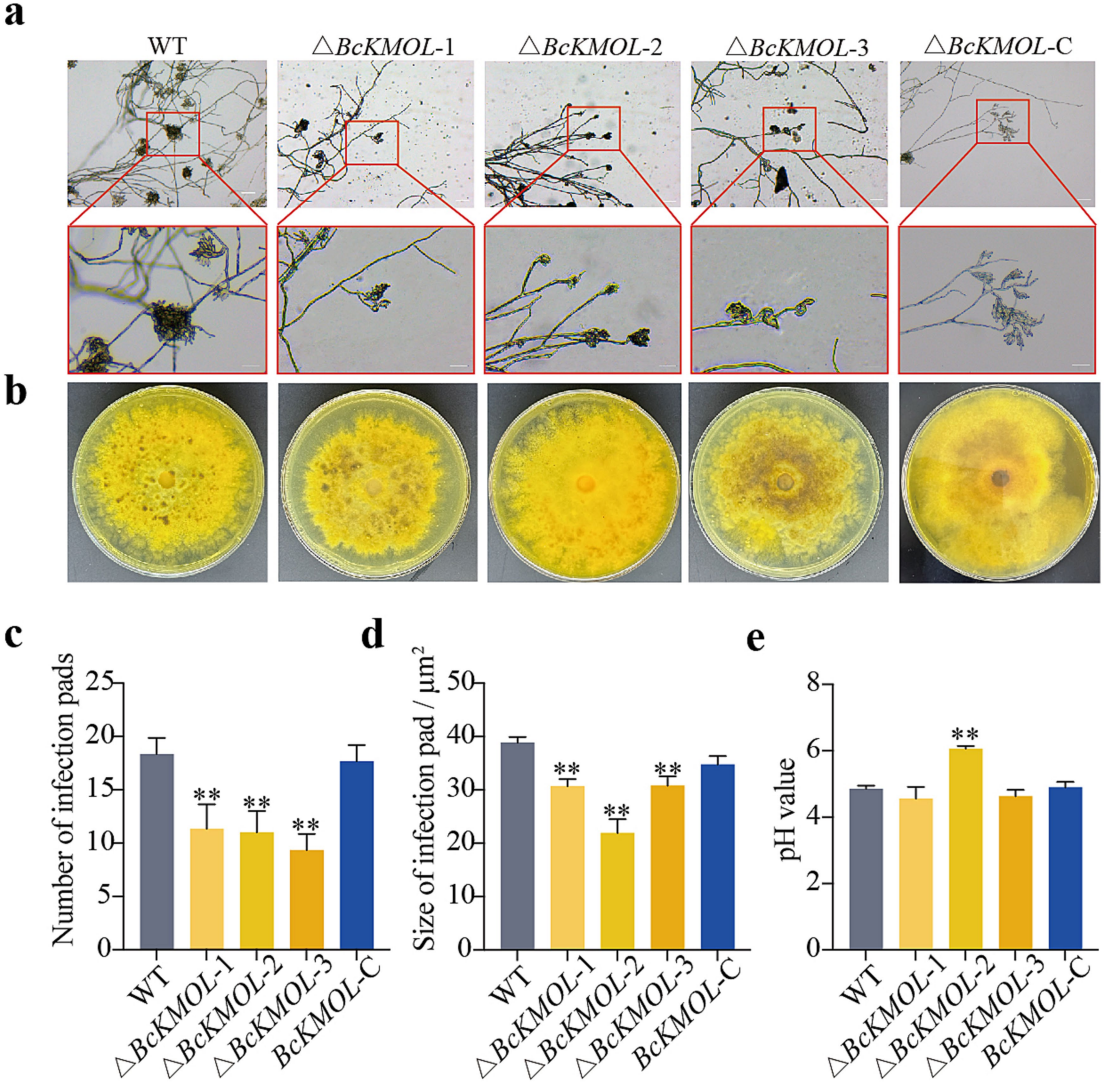
Infection cushion formation and acid production ability. **(a)** Infection cushion observation. Infection cushion formed during the same growth period were examined by microscopy. Scale bar: 10 μm. **(b)** Acid production analysis. Mutants were inoculated on the medium containing bromothymol blue to observe color change, indicating pH changes due to acid production. **(c)** The number of infection cushions of *BcKMOL* gene mutants. **(d)** The statistics of *BcKMOL* gene mutant’s infection cushion size. **(e)** The pH value of *BcKMOL* gene mutants. Each histogram represents the average SD from three biological replicates, and the asterisk indicates a significant difference from WT, *p < 0.05, **p < 0.01. Error bars represent the standard deviation from three biological replicates. Asterisks indicate significant differences from the wild type (WT); *p < 0.05, **p < 0.01.

As a necrotrophic plant pathogenic fungus, *B. cinerea* develops multicellular structures known as infection cushions specifically for plant penetration. To elucidate the role of the *BcKMOL* gene in the pathogenic process of this fungus, we analyzed the infection cushions and acid production capabilities of *B. cinerea* wild-type, along with *BcKMOL* gene mutants (Figure 4). The results showed that the number and size of the infection cushions of the △*BcKMOL* mutants were significantly different from those of the wild type and the revertant mutant (Figure 4). Although the colony color of the △*BcKMOL* mutants was largely similar to that of the wild type, we further assessed the colony’s pH value and found no significant difference between the *BcKMOL* mutant and the wild type. These results suggest that the *BcKMOL* gene negatively regulates the formation of infection cushion in *B. cinerea*.

### 3.5 *BcKMOL* influences the activity of cell wall degrading enzymes in *B. cinerea*

**Figure 5:**
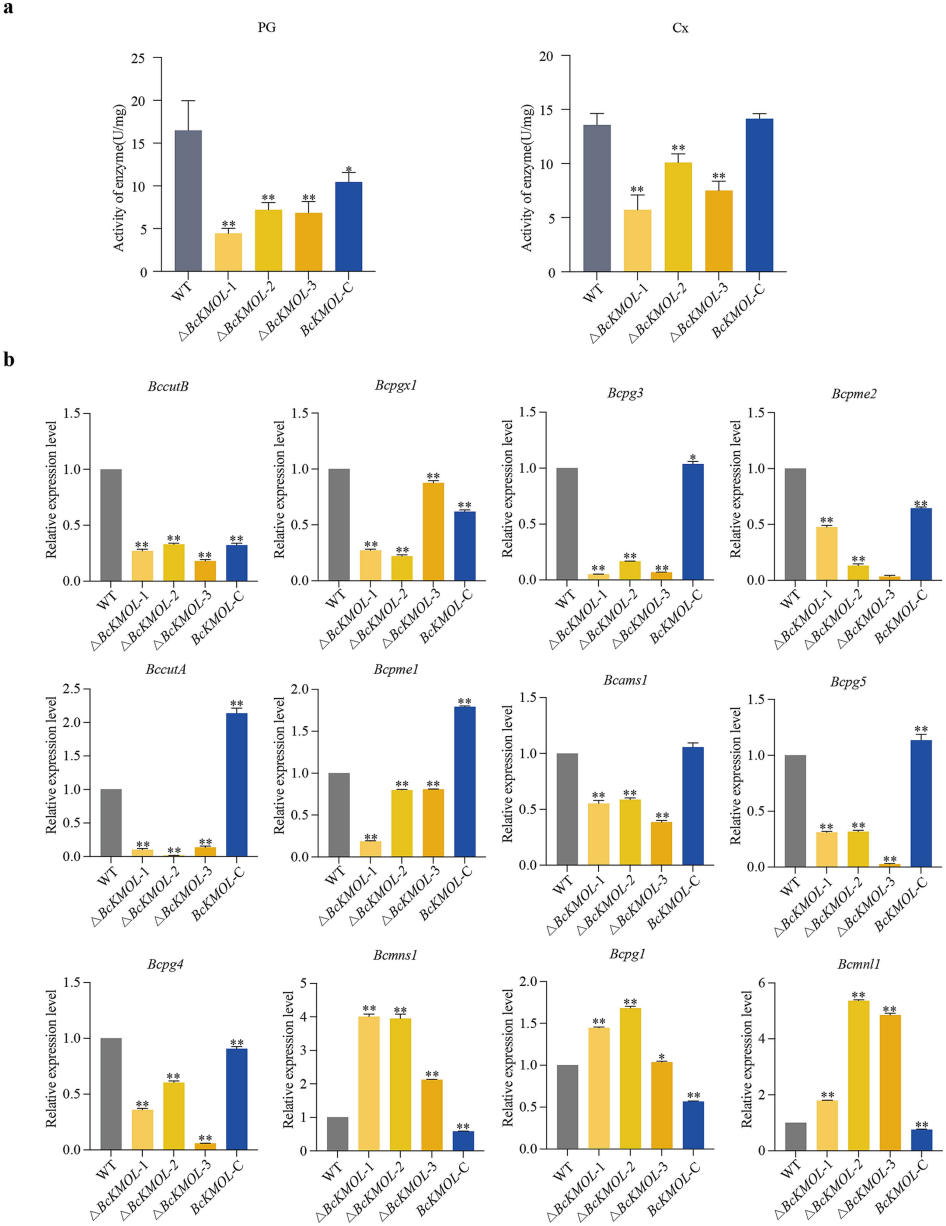
Analysis of pathogenic factors. **(a)** Extracellular enzyme activities. Pectinase (PG) and cellulase (Cx) activities were determined in the *BcKMOL* gene mutants and wild-type strains after 14 days of growth in liquid PD medium. **(b)** Analysis of expression levels of cell wall degrading enzyme-related genes. The *BcKMOL* gene mutants and wild-type strains at the same growth period were taken, and the cDNA was extracted. The cell wall degrading enzyme-related genes were detected by qRT-PCR. *Tubulin* served as the internal reference gene. Error bars represent the standard deviation from three biological replicates. Asterisks indicate significant differences from the wild type (WT); *p < 0.05, **p < 0.01.

The host infection by fungi primarily depends on cell wall permeability and integrity, which are governed by the activity of cell wall degrading enzymes (Zhang et al., 2009). Consequently, we assessed the extracellular pectinase and cellulase activities in the △*BcKMOL* mutants. Our results indicated that the deletion of *BcKMOL* led to a marked reduction in the extracellular pectinase and cellulase activities of the pathogen, whereas these activities were restored in the *BcKMOL*-C strain (Figure 5a). Additionally, we examined the expression levels of genes associated with cell wall degradation enzyme activity (Table 1). We observed significant down-regulation in the expression of *BccutB*, *Bcpgx1*, *Bcpg3*, *Bcpme2*, *BccutA*, *Bcpme1*, *Bcams*, *Bcpg5,* and *Bcpg4*. Conversely, only the expression levels of *Bcpg1*, *Bcmns,* and *Bcmnl* were up-regulated (Figure 5b).

### 3.6 Antibacterial peptide targeting *BcKMOL* can effectively suppress the pathogenicity of *B. cinerea*

We utilized *BcKMOL* as the bait protein and employed a multi-step screening process involving MEGADOCK for rough screening, HDOCK for fine screening, and AlphaFold3 for one-to-one nuanced analysis. This approach allowed us to identify 20 potential candidates. Notably, the iptm + ptm values for the bait protein and the library proteins with IDs of CAMPSQ3966 and CAMPSQ4589 exceeded 0.75. Remarkably, the iptm + ptm value for the CAMPQ3966 protein reached 1.49, suggesting a very high probability of interaction with the bait protein (Table 2).

**Table 2:** Screening antimicrobial peptides with KMO as bait protein.

Library protein ID	Docking score	Plddt	Iptm	Ptm	Iptm + ptm
CAMPSQ3966	−355.03	81.44258984	0.69	0.8	1.49
CAMPSQ4589	−322.31	83.86399627	0.19	0.65	0.84

The pTM value is the predicted TM score, which is used to evaluate the prediction accuracy of the overall protein three-dimensional structure. This index predicts the correctness of the overall folding of the protein and compares it with the similarity of the experimental structure; ipTM is the interface prediction TM score, which is used to evaluate the prediction accuracy of two interaction interfaces in the protein complex. It specifically evaluates the accuracy of structural prediction of contact regions between two or more proteins.

**Figure 6:**
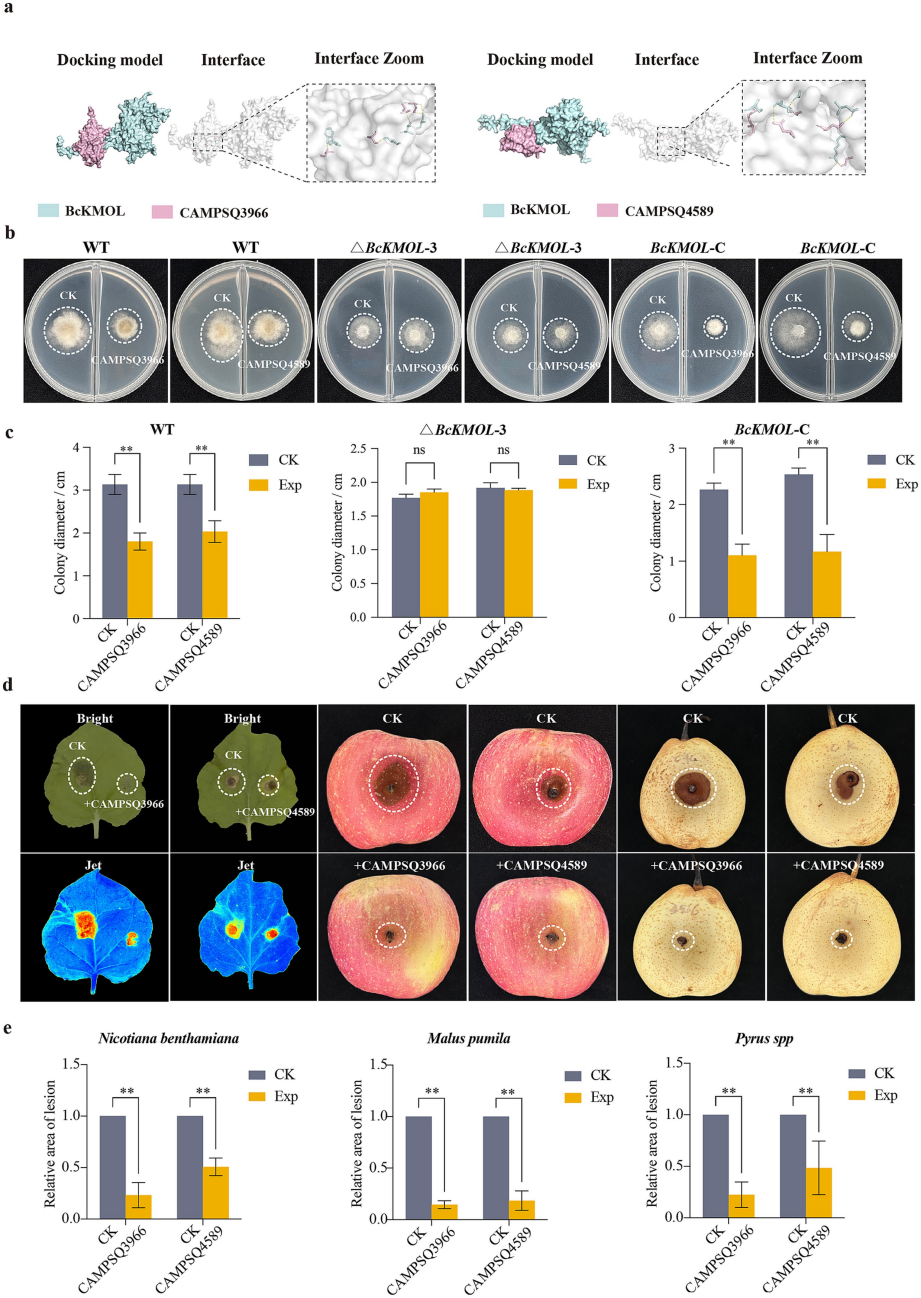
Screening and disease resistance analysis of antimicrobial peptides targeting *BcKMOL*. **(a)** Molecular docking simulation. Three-dimensional structures of *BcKMOL*, CAMPQ3966, and CAMPQ4589 were modeled using the Swiss-Model, and docking was performed using HDOCK. Results were analyzed in Pymol for interaction sites. **(b)** The binding analysis of antimicrobial peptides CAMPSQ3966 and CAMPSQ4589 with *BcKMOL*. The antibacterial peptides CAMPSQ3966 and CAMPSQ4589 of the same concentration were evenly coated on the right side of the culture dish, and the left side was coated with an equal volume of protein eluent as a control group. Wild type, △*BcKMOL*, and *BcKMOL*-C were inoculated, respectively. **(c)** The growth statistics of *BcKMOL* mutant colonies treated with antibacterial peptides CAMPSQ3966 and CAMPSQ4589. The diameter of colony growth at 48 hpi was recorded. **(d)** The disease resistance analysis of antimicrobial peptides CAMPSQ3966 and CAMPSQ4589. The protein concentration of CAMPSQ3966 and CAMPSQ4589 was adjusted to be consistent. The protein elution buffer was applied to the left side of the tobacco, and the proteins CAMPSQ3966 and CAMPSQ4589 were applied to the right side of the tobacco leaves, respectively. After dark treatment for 12 hpi, the wild-type strain of *Botrytis cinerea* at the same growth period was inoculated; the proteins CAMPSQ3966 and CAMPSQ4589 were mixed with Tween in equal proportions, and the protein eluent was mixed with Tween in equal proportions in the control group. The wild-type strains of *Botrytis cinerea* at the same growth period were inoculated and placed in the dark to observe the incidence. The statistics of lesion area after treatment of crops with antimicrobial peptides CAMPSQ3966 and CAMPSQ4589. Each histogram represents the average SD from three biological replicates, and the asterisk indicates a significant difference from WT, *p < 0.05, **p < 0.01.

The binding sites of CAMPQ3966 and CAMPQ4589 to *BcKMOL* were subjected to further analysis using HDOCK and Pymol (Figure 6a). Specifically, the binding sites of CAMPQ3966 to *BcKMOL* encompassed THR (82)–THR (469), THR (85)–SER (466), SER (148)–ILE (472), and SER (184)–TRP (483). For CAMPQ4589 binding to *BcKMOL*2, the sites included ASN (8)–VAL (467), ASN (27)–LEU (419), SER (31)–GLY (463), GLY (56)–ARG (429), and GLN (143)–SER (470). Expression studies revealed that CAMPQ3966 could be produced in abundance at 16°C, while CAMPQ4589 was expressed in large quantities at 28°C and subsequently purified using an AKTA protein purification instrument (Supplementary Figures S4a,b). The wild type and *BcKMOL* mutants were treated with antimicrobial peptides, and it was found that the wild type and *BcKMOL*-C were sensitive to the antimicrobial peptides CAMPSQ3966 and CAMPSQ4589, and the growth was significantly inhibited, while the growth of △*BcKMOL* did not change significantly (Figures 6b,c), which indicated that the antimicrobial peptides CAMPSQ3966 and CAMPSQ4589 acted on *BcKMOL*.

The purified antimicrobial peptides CAMPSQ396 and CAMPSQ4589 were applied to the right side of the tobacco leaves, and the blank control was set on the left side. The results showed that the leaves smeared with CAMPSQ3966 or CAMPSQ4589 had a significant inhibitory effect on the infection of *B. cinerea*. After treatment with antimicrobial peptides, the lesion area of the same apple or pear was significantly lower than that of the control group (Figures 6d,e), and the biomass of *B. cinerea* at the lesion of the leaves was detected. It was found that the biomass of *B. cinerea* was significantly reduced after treatment with CAMPSQ3966 or CAMPSQ4589 (Supplementary Figure S4c). It is indicated that the antibacterial peptides CAMPSQ396 and CAMPSQ4589 can inhibit the growth of *B. cinerea* and inhibit the pathogenicity of the pathogen.

## 4 Discussion

Kynurenine monooxygenase KMO, as a key rate-limiting enzyme in the kynurenine pathway (Xue et al., 2023), has become an important target for the treatment of neurodegenerative diseases in the human body. During the metabolic process, it can produce a series of neurotoxic substances such as kynurenine, 3-hydroxykynurenine and the neuroprotective substance kynurenic acid, and the imbalance of these metabolite levels is the key to the occurrence of the disease (Schwarcz et al., 2012). For example, it has been reported that KMO activity is up-regulated in the brain region of Huntington’s disease model mice (Sathyasaikumar et al., 2010). By inhibiting KMO activity, KP metabolic imbalance can be normalized, which can improve disease-related phenotypes (Bondulich et al., 2021). In this study, we detected the accumulation of tryptophan Trp content and the decrease of neurotoxic substances kynurenine KYNU, 3-hydroxykynurenine 3-HK, and quinolinic acid QA after the deletion of the *BcKMOL* gene in *Botrytis cinerea*. We speculated that the changes in these metabolite levels affected the growth and development and pathogenic process of the pathogen. Therefore, we analyzed the growth and development of *BcKMOL* gene knockout mutants. The most obvious is that the mutant △*BcKMOL* does not produce sclerotia, while the sclerotium structure of *Botrytis cinerea* is composed of compact vegetative mycelium cells and nutrients. It belongs to asexual resting structure (Elad et al., 2007; Richardson et al., 2011). Sclerotia can survive under various harsh conditions, such as low temperature or high temperature, drying and extreme lack of nutrients (Bolton et al., 2006). The pathogenicity of the mutant △*BcKMOL* was weakened. We analyzed the factors affecting the pathogenicity of the pathogen, such as the growth of the infection pad and the acid production ability of the pathogen. The size and number of the infection pad of the *BcKMOL* mutants were significantly affected, while *Botrytis cinerea* mainly penetrates the epidermis directly through the infection pad (Backhouse and Willets, 1987; Kunz et al., 2006; Sharman and Heale, 1977) or infects plants through wounds (Williamson et al., 2007). At the same time, several studies have shown that the main factors affecting the host ‘s resistance to fungal infection are cell wall permeability and integrity, which are maintained by the activity of cell wall degrading enzymes (Zhang et al., 2009). After knocking out the *BcKMOL* gene, we found that the activities of pectinase and cellulase were significantly down-regulated, and the expression levels of most genes related to cell wall degrading enzymes were down-regulated. Therefore, we can determine that *BcKMOL* plays an important role in the growth, development and pathogenicity of the pathogen. It is speculated that the *BcKMOL* gene deletion mutant of *Botrytis cinerea* may be due to changes in the level of kynurenine pathway metabolites affecting the growth, development, and infection structure of the pathogen, thereby affecting the pathogenic process of the pathogen. In the *BcKMOL* deletion mutant, we not only detected a decrease in neurotoxic substances but also a decrease in the neuroprotective substance KYNA. Therefore, the next step we will focus on the dynamic balance of the key metabolites of the kynurenine pathway and the ratio of key metabolites to help us better intervene in the pathway.

In recent years, soluble KMOs involved in kynurenine pathway metabolism have been identified in many bacteria, such as *P. fluorescens* (Kurnasov et al., 2003), *Cytophaga hutchinsonii* (Crozier and Moran, 2007) and *Ralstonia metallidurans* (Kurnasov et al., 2003). With the clarification of KMO crystal structure and detailed kinetic studies, the development of KMO inhibitors has been accelerated (Amaral et al., 2013), and a series of new compounds leading to the inhibition of KMO activity have been identified (Zhang et al., 2021) For example, the elevation of 3-hydroxykynurenine in mice can cause death from acute pancreatitis, and blocking the pathway with KMO-specific inhibitor GSK898 can reduce the critical phenotype and death (Hayes et al., 2023); oral administration of Ro61-8048 in mice can inhibit KMO and increase the concentration of kynurenic acid KYNA in the brain (Zwilling et al., 2011). KMO can be used as the basis for virtual screening by using the data of structure and ligand interaction, and combined with prodrug strategy to develop permeable KMO inhibitors (Zhang et al., 2019). After determining that *BcKMOL* positively regulates the growth, development and pathogenicity of *B. cinerea*, we used MEGADOCK, HDOCK, and AlphaFold3 to screen the potential antifungal peptides of *BcKMOL*. The results showed that the antifungal peptides CAMPSQ3966 and CAMPSQ4589 could effectively inhibit the growth and pathogenesis of *B. cinerea*. In recent years, the abuse of fungicides against *Botrytis cinerea* has led to increased crop resistance and increasingly serious pesticide residues. Antimicrobial peptides (AMPs) are considered to be a new generation of antibiotics, which have strong antibacterial activity against various bacteria, fungi, and viruses (Chung and Khanum, 2017; Mygind et al., 2005). In addition, AMP also has the functions of anti-biofilm and immune regulation (Luo and Song, 2021), and AMP has the advantages of low toxicity, strong thermal stability, high solubility, low molecular weight and no resistance to eukaryotic cells (Li et al., 2019). It has been reported in the literature that the soybean peptide sequence with high anti-inflammatory effect has been successfully identified, and it has been determined that the sequence of the antibacterial peptide with high activity is characterized by hydrophobic amino acids at the N-terminus and alkaline amino acids at the C-terminus (Liu et al., 2025). In this study, the antibacterial peptides obtained by purification and screening were found by pathogenicity test. CAMPSQ3966 and CAMPSQ4589, the antibacterial peptides of *BcKMOL*2, can effectively inhibit the pathogenicity of *Botrytis cinerea*. It provides a valuable exploration path for the follow-up study of the mechanism of KMO regulating the kynurenine pathway and thus affecting the pathogenicity of the pathogen. However, the length of the antimicrobial peptide CAMPQ3966 is 222 amino acids, and the length of CAMPQ4589 is 148 amino acids. Due to the length of the peptide and the cumbersome process of prokaryotic expression and purification, it is impossible to achieve its industrial production. However, in the future, we can combine the molecular docking results with the KMO crystal structure to analyze the key sites of the antimicrobial peptide and the target protein *BcKMOL*, and determine the active core region of the antimicrobial peptide, which provides a new idea for the safe, effective and long-term prevention and treatment of gray mold.

## 5 Conclusion

*BcKMOL*, which encodes kynurenine monooxygenase, lays a crucial role in the growth, development, and pathogenicity of *B. cinerea*. The *BcKMOL* mutation affects the metabolites of the kynurenine pathway and regulates the pathogenic process of the pathogen by regulating the formation of sclerotia, mycelial and spore morphology, infection cushion development, acid production capacity and cell wall degradation enzyme activity. In this study, we screened two antimicrobial peptides, CAMPQ3966 and CAMPQ4589, that effectively inhibit the pathogenicity of *B. cinerea*. These findings establish a foundation for the functional analysis of the kynurenine pathway in *B. cinerea* and offer novel insights into exploring new drug targets for the safe prevention and control of gray mold.

## Data Availability

The original contributions presented in the study are included in the article/Supplementary material, further inquiries can be directed to the corresponding author.

## Footnotes

^1^ https://swissmodel.expasy.org

^2^ https://www.ncbi.nlm.nih.gov/geo/

^3^ https://swissmodel.expasy.org

^4^ http://hdock.phys.hust.edu.cn
