# Supplementary material for: Kynurenine monooxygenase BcKMOL: a key regulator of growth, pathogenicity, and disease control in Botrytis cinerea
Source: Front Microbiol. 2025 Jun 24;16:1595008. doi: 10.3389/fmicb.2025.1595008 (PMC12237254; doi:10.3389/fmicb.2025.1595008)
Supplement: Supplementary file 1 [file Table_1.docx]

Supplementary Table 1 The primers sequence.

| Gene name | Primer name | Sequence（5’ - 3’） |
| --- | --- | --- |
| *BcKMOL*Ⅰ | *BcKMOL*Ⅰ-F | GAGCTCTGTTTGGTACGGCTTTAGAAT |
|  | *BcKMOL*Ⅰ-R | ACTAGTGCGAGGAACTCCATCTCTCCA |
| *BcKMOL*Ⅱ | *BcKMOL*Ⅱ-F | CTCGAGCATAAGGGTCGTTAAATGAGC |
|  | *BcKMOL*Ⅱ-R | GGTACCTGTCAAGTCGAAGGAGCACA |
| pEarleyGate104-BcKMOL | pEarleyGate104-BcKMOL-F | CACCATGTCTGGACAATCTTCAAGACA |
|  | pEarleyGate104-BcKMOL-R | AGCAGTCTTCCATCCCAATAATGCT |
| *BcKMOL*  hygromycin B | P1 | CAACATGTTTGAAGCTTGGCAC |
|  | P2 | GGAGCAGCAGACGCGCTA |
| *BcKMOL*  hygromycin B | P3 | CTGCAGAACAGCGGGCAG |
|  | P4 | GACATGTTCATCCTAGACAATTGG |
| hygromycin B | P5 | CTATTCCTTTGCCCTCGGA |
|  | P6 | ATGAAAAAGCCTGAACTCACCGC |
| *BcKMOL* | P7 | TCATCCCCTCGTTGAGCTG |
|  | P8 | ATTGTAAAGAAGAGCCAGAAACAGG |
| Rt*BcKMOL* | Rt*BcKMOL*-F | CGACACTCTGTGGTGTGAGT |
|  | Rt*BcKMOL*-R | TCGAGCTCATTGAACGGCTT |
| KanR | KanR-F | CTCCCAATCAGGCTTGATCCC |
|  | KanR-R | ATGGCTAAAATGAGAATATCACCGG |
| GLF | GLF-F | TCAAATCTGGTGACGGGCAGGAC |
|  | GLF-R | ATGAGCCCAGAACGACGC |
| *Bcpg1* | *Bcpg1*-rt-F | ATGGTTCAACTTCTCTCAATGGC |
|  | *Bcpg1*-rt-R | TGGGACGGAGAGTGCGCTGAGGACG |
| *Bcpg5* | *Bcpg5*-rt-F | ATGGTTAAGTTTTCTGCCTGTC |
|  | *Bcpg5*-rt-R | GCCAGATGGGACTGCAAG |
| *Bcpgx1* | *Bcpgx1*-rt-F | ATGCATTTTCAATTGAGC |
|  | *Bcpgx1*-rt-R | GGTTCTAGCTGCCGGAGAAGGTGCA |
| *Bcpg4* | *Bcpg4*-rt-F | ATGCCTTCCACCAAGTCCA |
|  | *Bcpg4*-rt-R | GATCGGTCATGTCAAGGGTGACACC |
| *Bcpg3* | *Bcpg3*-rt-F | ATGCGTTCTGCGATCATCCTC |
|  | Bcpg3-rt-R | TTGAAGACAACTGCATGCACTAGAC |
| *Bcpme2* | *Bcpme2*-rt-F | ATGCGTTCCTTTGCCCTCCTCTCCC |
|  | *Bcpme2*-rt-R | ATCGGTGGTGGTGGTGGACAATGCA |
| *Bcmnl1* | *Bcmnl1*-rt-F | ATGGAGCTGGCATTCCCGGA |
|  | *Bcmnl1*-rt-R | TTGAAGTGCTGTTGCGCCGGTTTCT |
| *Bcams1* | *Bcams1*-rt-F | ATGGGTGGTGAAACTGTCTTA |
|  | *Bcams1*-rt-R | CGATAGTTTGATGTGCGGTTCTCCA |
| *Bcpme1* | *Bcpme1*-rt-F | ATGCCGCAGTTCAGAGGAAGCCC |
|  | *Bcpme1*-rt-R | TTGGTATCCGATGAACTGGGTGGCA |
| *BccutA* | *BccutA*-rt-F | ATGAAGACCTCAGCTCAACAAC |
|  | *BccutA*-rt-R | GGTGACTGAAGATGACCCAAGAGCA |
| *BccutB* | *BccutB*-rt-F | ATGAAGTTTTCACAGTCTCTG |
|  | *BccutB*-rt-R | GGCAAAGATAAGCATCATTGGTGCG |
| *Bcmns1* | *Bcmns1*-rt-F | ATGAACAGTGCGACCCCTTTAACC |
|  | *Bcmns1*-rt-R | CAAATTGCGCTGAGGATCTGTGAAA |
| *β-Tublin* | *β-Tublin*-F | GAGCGTGAAATCGTCCGTG |
|  | *β-Tublin*-R | GGATACCACCGCTCTCAAGAC |
