# Supplementary figures and images for: Kynurenine monooxygenase BcKMOL: a key regulator of growth, pathogenicity, and disease control in Botrytis cinerea
Source: Front Microbiol. 2025 Jun 24;16:1595008. doi: 10.3389/fmicb.2025.1595008 (PMC12237254; doi:10.3389/fmicb.2025.1595008)

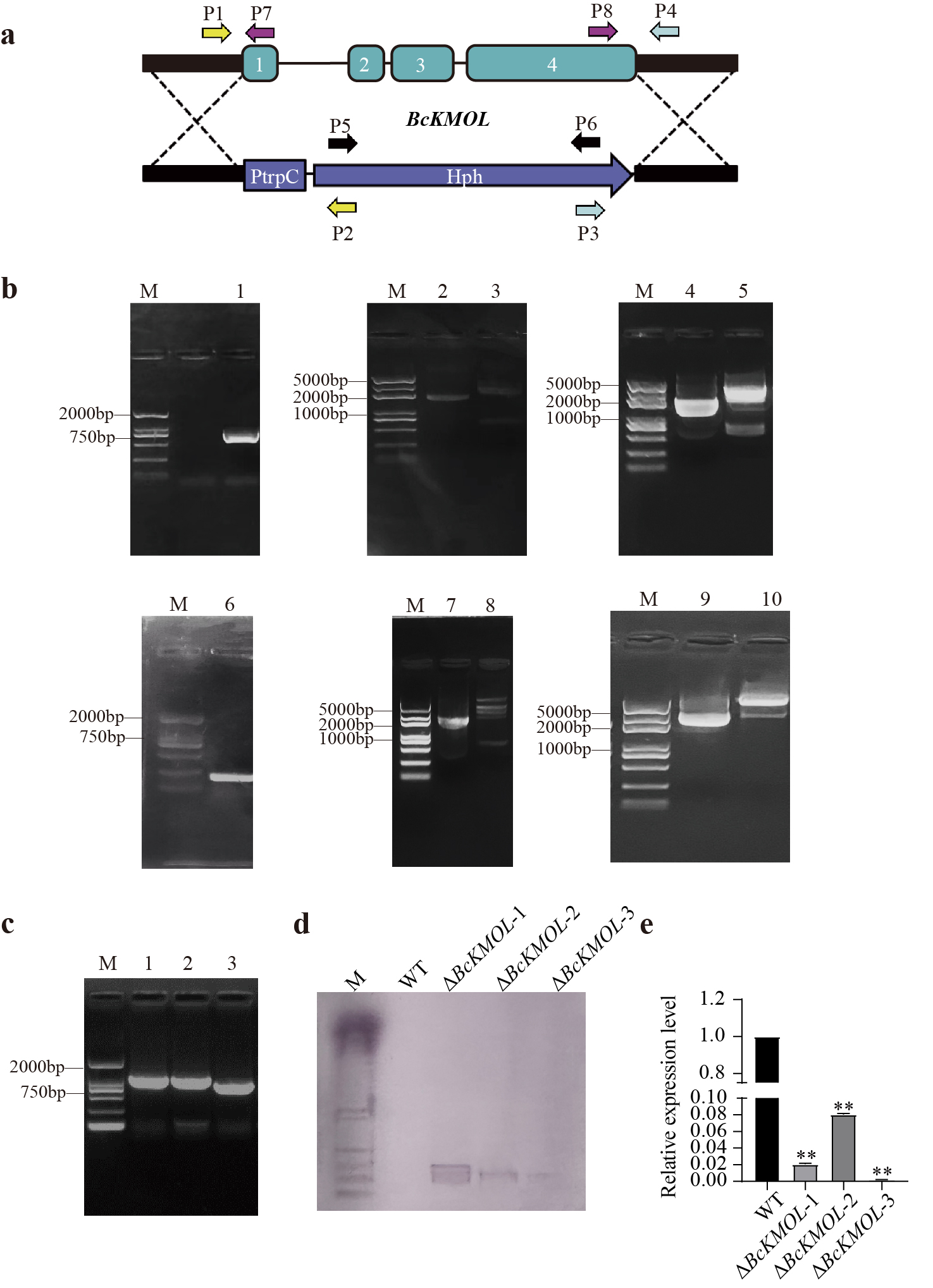

Supplement: Supplementary file 2 [file Image_1.jpeg]

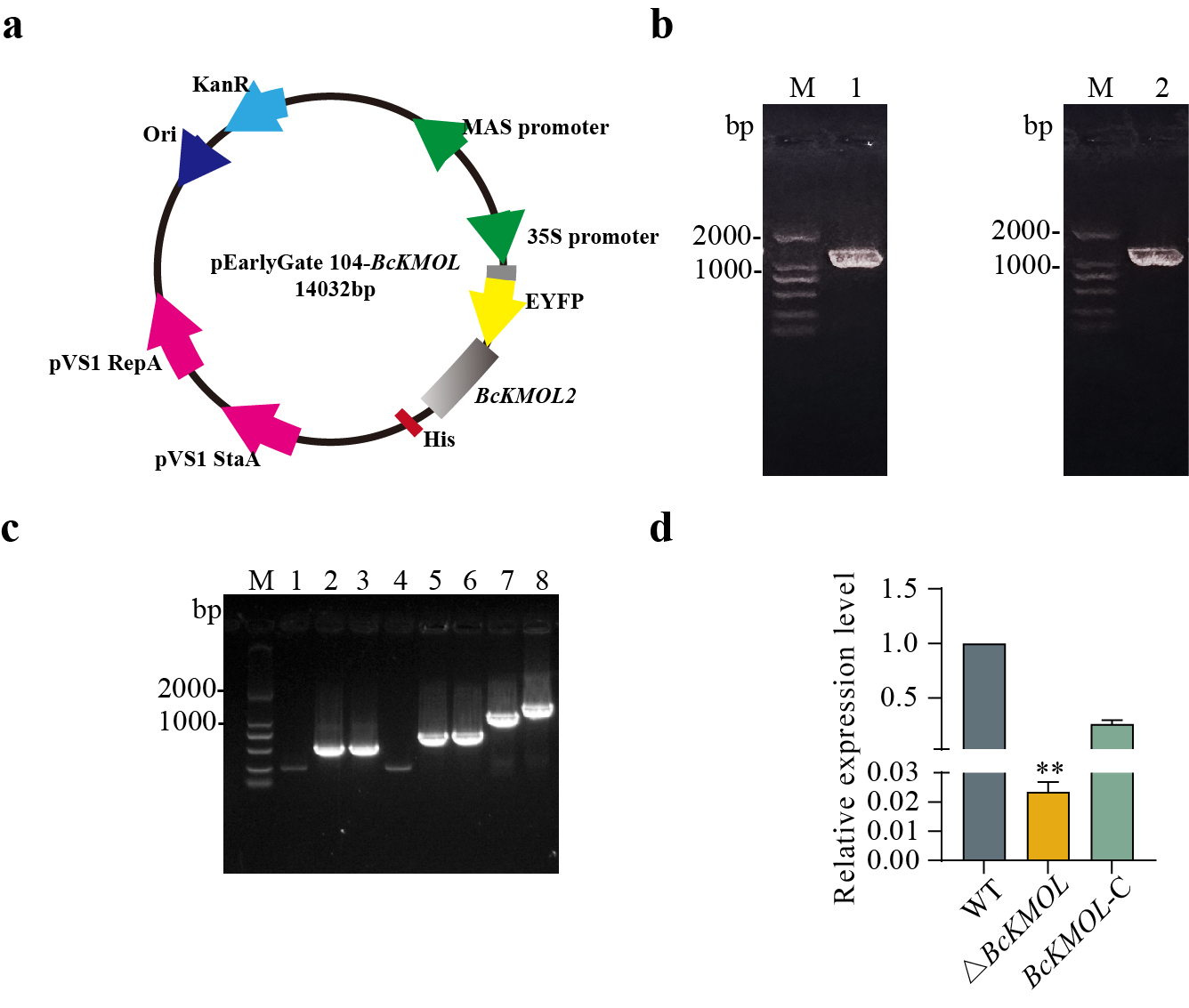

Supplement: Supplementary file 3 [file Image_2.jpeg]

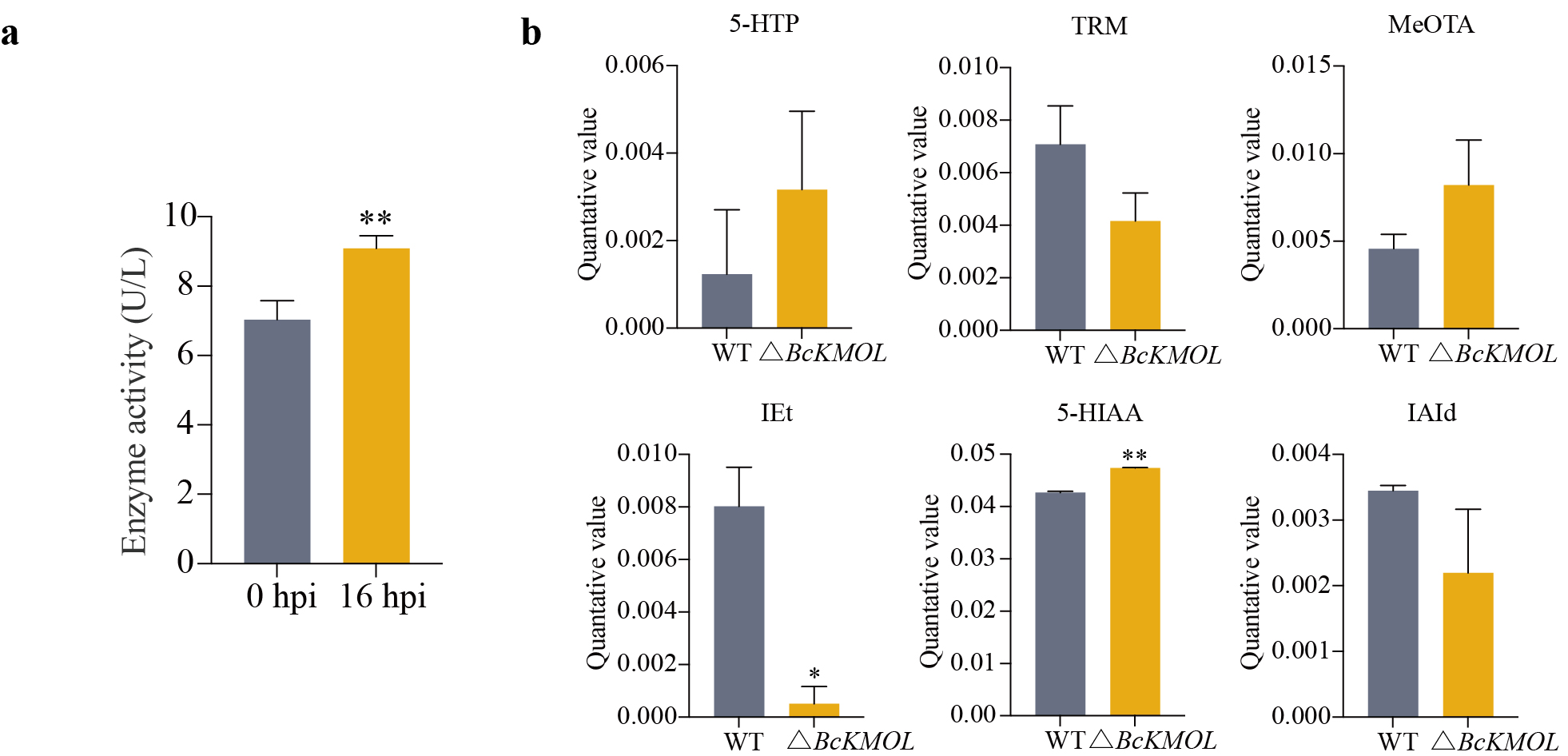

Supplement: Supplementary file 4 [file Image_3.jpeg]

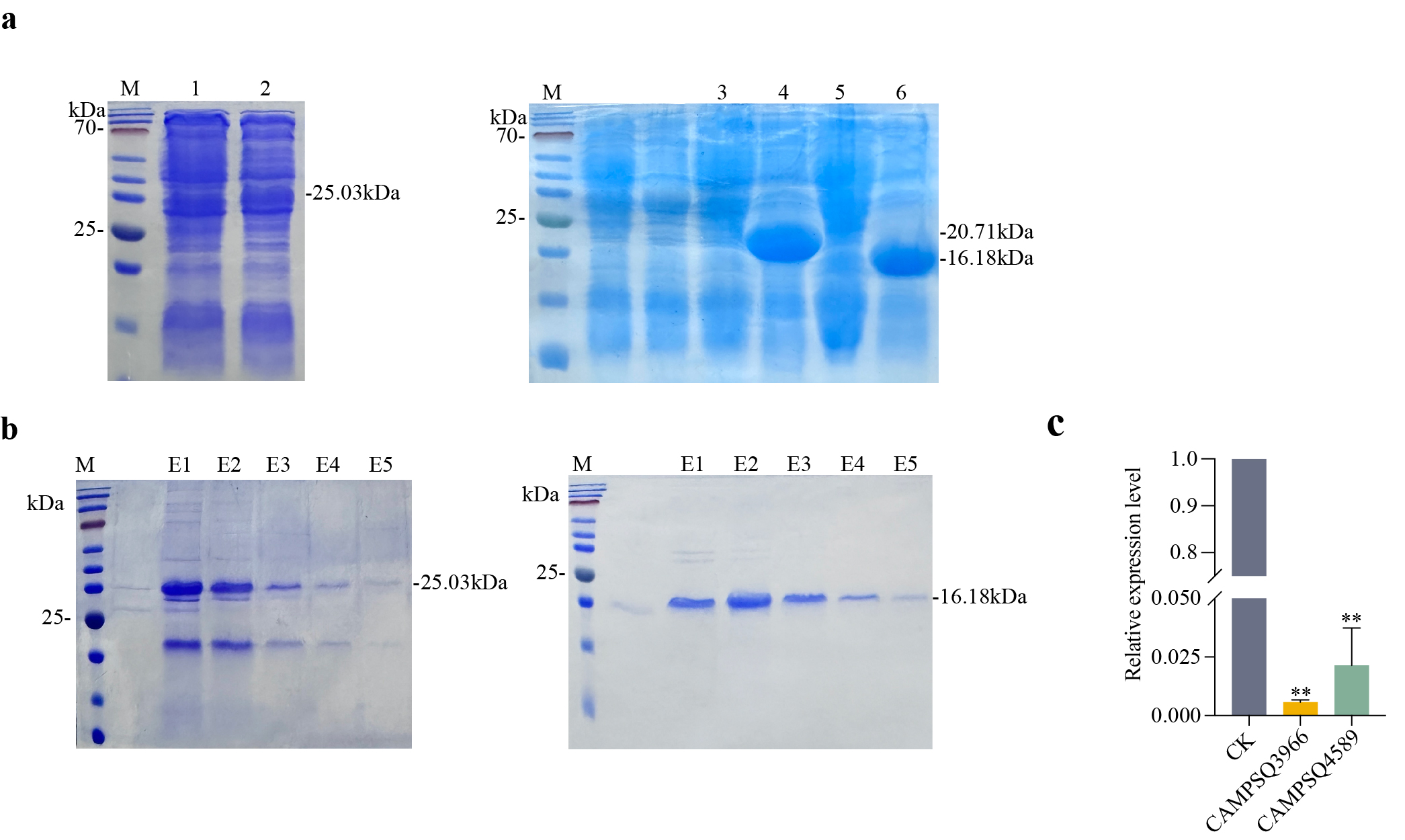

Supplement: Supplementary file 5 [file Image_4.jpeg]
